# Supplementary material for: Online randomised trials with children: A scoping review
Source: PLoS One. 2023 May 25;18(5):e0280965. doi: 10.1371/journal.pone.0280965 (PMC10212186; doi:10.1371/journal.pone.0280965)
Supplement: S3 Appendix — (ZIP) [file pone.0280965.s006.zip › S7_Appendix/Hillhouse.docx]

| **Basic Study Characteristics** | |  |  |  |  |  |  |
| --- | --- | --- | --- | --- | --- | --- | --- |
| **Citation** | Hillhouse J, Turrisi R, Scaglione N, Cleveland M, Baker K, Florence L, et al. A Web-Based Intervention to Reduce Indoor Tanning Motivations in Adolescents: a Randomized Controlled Trial. Prevention Science. 2017;18(2):131-40. doi: 10.1007/s11121-016-0698-4. PubMed PMID: 120784944. Language: English. Entry Date: 20180723. Revision Date: 20191107. Publication Type: journal article. |  |  |  |  |  |  |
| **Characteristic reported (indicate with X for decision or enter free text, where applicable)** | |  |  |  |  |  |  |
| Type of study | \| Randomised trial \| Cluster randomised \| \| --- \| --- \| \| Quasi-randomised trial \| Individually randomised \| |  |  |  |  |  |  |
| Type of publication | \| Peer-reviewed journal \| \| --- \| \| Pre-print \| \| Other, if so, describe \| |  |  |  |  |  |  |
| Aim of study as per author’s description | To examine the efficacy of a web-based intervention to reduce tanning motivations in female adolescents. |  |  |  |  |  |  |
| **Participant Characteristics** | |  |  |  |  |  |  |
| Participant age demographics | \| Age range \| 12-18 \| \| --- \| --- \| \| Mean age \| 15.2 \| |  |  |  |  |  |  |
| Country(ies) of Participants’ Residence | U.S.A. |  |  |  |  |  |  |
| Number of participants randomised | \| Children 443 \| \| --- \| \| Caregivers Not reported \| |  |  |  |  |  |  |
| Gender demographics of participants | 100% female |  |  |  |  |  |  |
| Socioeconomic status of participants | Not reported |  |  |  |  |  |  |
| Race/Ethnicity of participants | From ClinicalTrial.gov: 12.2% Hispanic or Latino  87.8% Not Hispanic or Latino |  |  |  |  |  |  |
| Type of participant targeted | Females |  |  |  |  |  |  |
| Children’s advisory group formation | \| Ages of advisory group participants \| Not reported \| \| --- \| --- \| \| Means of recruiting advisory group participants \| Not reported \| \| Phases of trial involvement \| Pilot test \|   Yes  No  Unclear  Not Applicable  Not Reported  (Was an advisory group formed in the planning/execution of the study?) |  |  |  |  |  |  |
| Inclusion/ Exclusion Criteria | Included: Parent-teen dyads with female daughters with previous indoor tanning use or strong intentions or willingness to indoor tan in the future |  |  |  |  |  |  |
| **Methods of Recruitment, Retention & Consent** | |  |  |  |  |  |  |
| Online methods/platforms used for recruitment | Recruited from Knowledge Networks KnowledgePanel, a commercial online panel for measurement of public opinion, attitudes and behaviours, uses probability-based sampling with an address-based sampling methodology for selecting panel members. |  |  |  |  |  |  |
| Offline methods used for recruitment | NA |  |  |  |  |  |  |
| Methods/tools used for online consent/assent acquisition | Parental and teen consent obtained |  |  |  |  |  |  |
| Caregiver consent acquired/ waived (If waived, by what authority?) | Yes  No  Unclear  Not Applicable  Not Reported  As above |  |  |  |  |  |  |
| Children assent acquisition | Yes  No  Unclear  Not Applicable  Not Reported |  |  |  |  |  |  |
| What, if any, methods were used to validate that assent/consent was informed? | Not reported  (E.g. validation questions, CAPTCHA, email confirmation, video-conferencing verbal) |  |  |  |  |  |  |
| Pushes/reminders sent to participants | \| Enrolment in trial  Yes  No  Unclear  Not Applicable  Not Reported \| \| --- \| \| Retention/completion in trial  Yes  No  Unclear  Not Applicable  Not Reported \| \| Follow-up in trial  Yes  No  Unclear  Not Applicable  Not Reported \| |  |  |  |  |  |  |
| **Interventions** | |  |  |  |  |  |  |
| Number of arms | 2 |  |  |  |  |  |  |
| Intervention | Participants viewed a website designed to reduce indoor tanning motivations and increase sunless tanning willingness. |  |  |  |  |  |  |
| Comparison | Control participants viewed a teen-oriented alcohol prevention website. |  |  |  |  |  |  |
| Operating Systems/Devices required for participants’ engagement with trial | Not reported  (E.g. computer, phone, iOS, Windows, Android, iPhone, Facebook or Instagram account etc.) |  |  |  |  |  |  |
| Tools/software used for data protection processes | Not reported  (E.g. dedicated website, REDCap, institutional server etc.) |  |  |  |  |  |  |
| Tools/methods used for data collection | Individual survey links were sent to participating youth via email or text.  (E.g. data submitted via website, video-conferencing etc.) |  |  |  |  |  |  |
| Did caregivers participate/ contribute data to the trial? If so, describe. | Yes  No  Unclear  Not Applicable  Not Reported |  |  |  |  |  |  |
| Duration of intervention from first to final engagement | Unclear, but appears to be an SSI + 6-month follow-up survey  “Participants from two cohorts completed baseline assessments in May of 2011 and 2012.” Pg. 8 |  |  |  |  |  |  |
| Compensation offered to participants | Participants were offered up to $120 for completing all study requirements. |  |  |  |  |  |  |
| **Outcomes** | |  |  |  |  |  |  |
| Results relevant to this scoping review | NA  (E.g. satisfaction with online methods of trial, demographics of participants’ in relation to their outcomes especially in regarding online components, i.e., recruitment, retention, completion etc) |  |  |  |  |  |  |
| Baseline differences between completers & non-completers | “Analyses of baseline differences between participants who completed follow-up versus those who did not revealed no significant differences. Furthermore, examination of baseline differences in intervention participants who did not complete follow-up versus control participants who did not complete follow-up revealed no significant differences.” Pg. 8 |  |  |  |  |  |  |
| Attrition rates of randomised participants | Intervention: 214 dyads randomised, 182 completed 6-month FU: 15% attrition  Control: 229 dyads randomised, 206 completed 6-month FU: 10% attrition |  |  |  |  |  |  |
| Limitations of study as described by authors | The technology used (website) became quickly outdated (vs social media sites). The intervention only assessed the effects of one possible alternative, sunless tanning. |  |  |  |  |  |  |
| Funding source | \| Institutional, i.e. governmental, university \| Yes  No  Unclear  Not Applicable  Not Reported \| \| --- \| --- \| \| Private, i.e. pharmaceutical, software company \| Yes  No  Unclear  Not Applicable  Not Reported \| |  |  |  |  |  |  |
| Other aspects of study pertinent to online trial conception and execution | “A professional digital marketing company, Marketing Strategies, Inc., converted modules developed by the research team into a website, which was pretested with adolescents and then further refined using an interactive process involving the investigative team, the web development team and adolescent tanner beta testers.” Pg. 5 |  |  |  |  |  |  |

| Risk of Bias assessment  ([**https://handbook-5-1.cochrane.org/chapter_8/8_assessing_risk_of_bias_in_included_studies.htm**](https://handbook-5-1.cochrane.org/chapter_8/8_assessing_risk_of_bias_in_included_studies.htm)**)** | | | | |
| --- | --- | --- | --- | --- |
| Bias domain | Source of bias | Risk of bias | Support for judgment (use direct quotes where possible with explanatory comments) | Location in text or source (pg. number, figure, table etc.) |
| Selection bias | Random sequence generation | Low risk    High risk  Unclear risk | “The invitation was accepted by 443 dyads (77.6% participation rate) who were randomized into intervention (n = 214) and control (n = 229) groups.”  Comment: Insufficient information to assess risk. | Pg. 4, under Participants |
|  | Allocation concealment | Low risk    High risk  Unclear risk | Comment: Insufficient information to assess risk. | Not reported |
| Performance bias | Blinding of participants and personnel | Low risk    High risk  Unclear risk | Comment: Participants were not blinded as the outcome variables were willingness and intentions to indoor tan, willingness to sunless tan and measures of indoor tanning attitudes and beliefs. | Pg.1, under Abstract |
| Detection bias | Blinding of outcome assessment | Low risk    High risk  Unclear risk | Comment: There is insufficient information to make an assessment. | Not reported |
| Attrition bias | Incomplete outcome data | Low risk    High risk  Unclear risk | Comment: Intervention: 214 dyads randomised, 182 completed 6-month FU: 15% attrition  Control: 229 dyads randomised, 206 completed 6-month FU: 10% attrition | Pg. 16, Fig. 2 |
| Reporting bias | Selective reporting | Low risk    High risk  Unclear risk | Comment: Study protocol is not available but the pre-specified outcomes listed in the clinical trials register have been reported and additional measures were reported in the study. | Registered at ClinicalTrials.gov: [NCT01508013](https://clinicaltrials.gov/ct2/show/record/NCT01508013) |
| Other bias | Anything else, ideally pre-specified | Low risk    High risk  Unclear risk | Comment: No other bias detected | Not applicable |
