# Supplementary material for: Online randomised trials with children: A scoping review
Source: PLoS One. 2023 May 25;18(5):e0280965. doi: 10.1371/journal.pone.0280965 (PMC10212186; doi:10.1371/journal.pone.0280965)
Supplement: S1 Form — (DOCX) [file pone.0280965.s007.docx]

# **Supporting Form S4.** Data charting tool for “Online randomised trials with children: A scoping review.”

|  | |  |  |  |  |  |  |
| --- | --- | --- | --- | --- | --- | --- | --- |
| **Basic Study Characteristics** | |  |  |  |  |  |  |
| **Citation** |  |  |  |  |  |  |  |
| **Characteristic reported (indicate with X for decision or enter free text, where applicable)** | |  |  |  |  |  |  |
| Type of study | \| Randomised trial \| Cluster randomised \| \| --- \| --- \| \| Quasi-randomised trial \| Individually randomised \| |  |  |  |  |  |  |
| Type of publication | \| Peer-reviewed journal \| \| --- \| \| Pre-print \| \| Other, if so, describe \| |  |  |  |  |  |  |
| Aim of study as per author’s description |  |  |  |  |  |  |  |
| **Participant Characteristics** | |  |  |  |  |  |  |
| Participant age demographics | \| Age range \|  \| \| --- \| --- \| \| Mean age \|  \| |  |  |  |  |  |  |
| Country(ies) of Participants’ Residence |  |  |  |  |  |  |  |
| Number of participants randomised | \| Children \| \| --- \| \| Caregivers \| |  |  |  |  |  |  |
| Gender demographics of participants |  |  |  |  |  |  |  |
| Socioeconomic status of participants |  |  |  |  |  |  |  |
| Race/Ethnicity of participants |  |  |  |  |  |  |  |
| Type of participant targeted | (E.g. LQBTQ, disease-specific, racial/ethnic minority etc.) |  |  |  |  |  |  |
| Children’s advisory group formation | (Was an advisory group formed in the planning/execution of the study?)  Yes  No  Unclear  Not Applicable  Not Reported   \| Ages of advisory group participants \|  \| \| --- \| --- \| \| Means of recruiting advisory group participants \|  \| \| Phases of trial involvement \|  \| |  |  |  |  |  |  |
| Inclusion/ Exclusion Criteria |  |  |  |  |  |  |  |
| **Methods of Recruitment, Retention & Consent** | |  |  |  |  |  |  |
| Online methods/platforms used for recruitment |  |  |  |  |  |  |  |
| Offline methods used for recruitment |  |  |  |  |  |  |  |
| Methods/tools used for online consent/assent acquisition |  |  |  |  |  |  |  |
| Caregiver consent acquired/ waived (If waived, by what authority?) | Yes  No  Unclear  Not Applicable  Not Reported |  |  |  |  |  |  |
| Children assent acquisition | Yes  No  Unclear  Not Applicable  Not Reported |  |  |  |  |  |  |
| What, if any, methods were used to validate that assent/consent was informed? | (E.g. validation questions, CAPTCHA, email confirmation, video-conferencing verbal) |  |  |  |  |  |  |
| Pushes/reminders sent to participants | \| Enrolment in trial  Yes  No  Unclear  Not Applicable  Not Reported \| \| --- \| \| Retention/completion in trial  Yes  No  Unclear  Not Applicable  Not Reported \| \| Follow-up in trial  Yes  No  Unclear  Not Applicable  Not Reported \| |  |  |  |  |  |  |
| **Interventions** | |  |  |  |  |  |  |
| Number of arms |  |  |  |  |  |  |  |
| Intervention |  |  |  |  |  |  |  |
| Comparison |  |  |  |  |  |  |  |
| Operating Systems/Devices required for participants’ engagement with trial | (E.g. computer, phone, iOS, Windows, Android, iPhone, Facebook or Instagram account etc.) |  |  |  |  |  |  |
| Tools/software used for data protection processes | (E.g. dedicated website, REDCap, institutional server etc.) |  |  |  |  |  |  |
| Tools/methods used for data collection | (E.g. data submitted via website, video-conferencing etc.) |  |  |  |  |  |  |
| Did caregivers participate/ contribute data to the trial? If so, describe. | Yes  No  Unclear  Not Applicable  Not Reported |  |  |  |  |  |  |
| Duration of intervention from first to final engagement |  |  |  |  |  |  |  |
| Compensation offered to participants |  |  |  |  |  |  |  |
| **Outcomes** | |  |  |  |  |  |  |
| Results relevant to this scoping review | (E.g. satisfaction with online methods of trial, demographics of participants’ in relation to their outcomes especially in regarding online components, i.e., recruitment, retention, completion etc) |  |  |  |  |  |  |
| Differences between completers & non-completers |  |  |  |  |  |  |  |
| Attrition rates of randomised participants |  |  |  |  |  |  |  |
| Limitations of study as described by authors |  |  |  |  |  |  |  |
| Funding source | \| Institutional, i.e. governmental, university \| Yes  No  Unclear  Not Applicable  Not Reported \| \| --- \| --- \| \| Private, i.e. pharmaceutical, software company \| Yes  No  Unclear  Not Applicable  Not Reported \| |  |  |  |  |  |  |
| Other aspects of study pertinent to online trial conception and execution |  |  |  |  |  |  |  |

| Risk of Bias assessment  ([**https://handbook-5-1.cochrane.org/chapter_8/8_assessing_risk_of_bias_in_included_studies.htm**](https://handbook-5-1.cochrane.org/chapter_8/8_assessing_risk_of_bias_in_included_studies.htm)**)** | | | | |
| --- | --- | --- | --- | --- |
| Bias domain | Source of bias | Risk of bias | Support for judgment (use direct quotes where possible with explanatory comments) | Location in text or source (pg. number, figure, table etc.) |
| Selection bias | Random sequence generation | Low risk    High risk  Unclear risk |  |  |
|  | Allocation concealment | Low risk    High risk  Unclear risk |  |  |
| Performance bias | Blinding of participants and personnel | Low risk    High risk  Unclear risk |  |  |
| Detection bias | Blinding of outcome assessment | Low risk    High risk  Unclear risk |  |  |
| Attrition bias | Incomplete outcome data | Low risk    High risk  Unclear risk |  |  |
| Reporting bias | Selective reporting | Low risk    High risk  Unclear risk |  |  |
| Other bias | Anything else, ideally pre-specified | Low risk    High risk  Unclear risk |  |  |
