# Supplementary material for: Online randomised trials with children: A scoping review
Source: PLoS One. 2023 May 25;18(5):e0280965. doi: 10.1371/journal.pone.0280965 (PMC10212186; doi:10.1371/journal.pone.0280965)
Supplement: Attachment — Submitted filename: Comments.docx [file pone.0280965.s008.docx]

This study presented a research question in summarising the evidence in the online RCTs in children. However, many issues should be addressed first before the manuscript can be considered for publication, especially the research problem clarification. My suggestions are as below:

Introduction:

1. Please reconsider the research question and make it more clearly. What specific online RCT did the authors want to evaluate. Which type of the children did the authors want to focus on. What specific problems of children did the authors want to address. After that, please also rewrite the title and introduction.
2. As only RCTs or quasi experimental studies were included, why a scoping review was still conducted, not a systematic review or meta-analysis?

Methods:

1. After reclarifying the research problem, the authors should redefine the inclusion criteria of the studies
2. How about the quality appraisal process? Please add more details of ROB

Results:

1. The authors seemed to write this review as a wrong direction. Actually, for the recruitment methods, consent, data collection, compensation, loss to follow-up, and public and patient involvement (PPI) in online trials within paediatric populations, these could be summarised as the quality appraisal results, but should not be the main focus of a systematic review. The main results of the review involving RCTs should summarised the components of the target intervention, such as intervention typer, mode, frequency, and the efficacy of the interventions.
2. What facilitators and gaps did the authors find out?

Discussion:

1. The authors should reconsider the significance of the findings for international scholars.
